# Supplementary material for: Increased Pathway Complexity Is a Prognostic Biomarker in Metastatic Castration-Resistant Prostate Cancer
Source: Cancers (Basel). 2021 Mar 30;13(7):1588. doi: 10.3390/cancers13071588 (PMC8037684; doi:10.3390/cancers13071588)
Supplement: Supplementary file 1 [file cancers-13-01588-s001.pdf]

Supplementary Materials: Increased pathway complexity is a prognostic biomarker in metastatic castration-resistant prostate cancer

Bram De Laere, Alessio Crippa, Ashkan Mortezavi, Christophe Ghysel, Prabhakar Rajan, Martin Eklund, Alexander Wyatt, Luc Dirix, Piet Ost, Henrik Grönberg, Johan Lindberg, on behalf of the CORE and ProBio Investigators

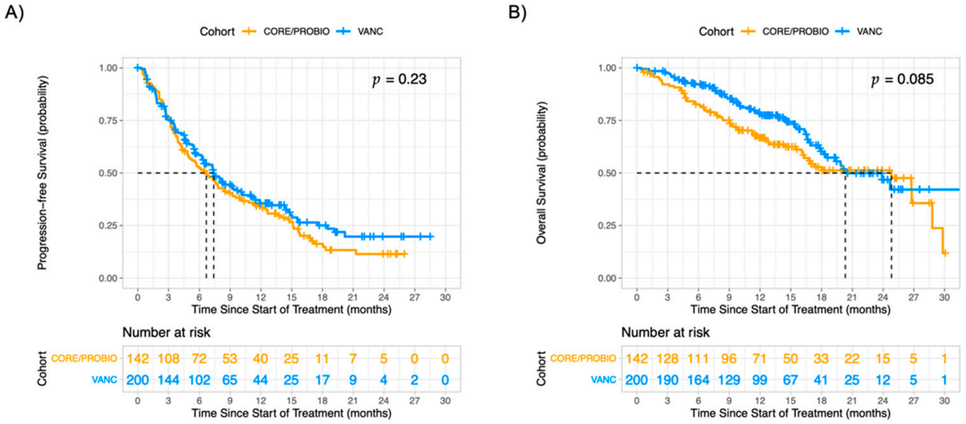

Figure S1. Kaplan-Meier analysis of progression-free survival **(A)** and overall **(B)** survival, stratified according to the study cohort, i.e. CORE/PROBIO (n = 142) and VANC (n = 200). p-value is calculated via log-rank test.

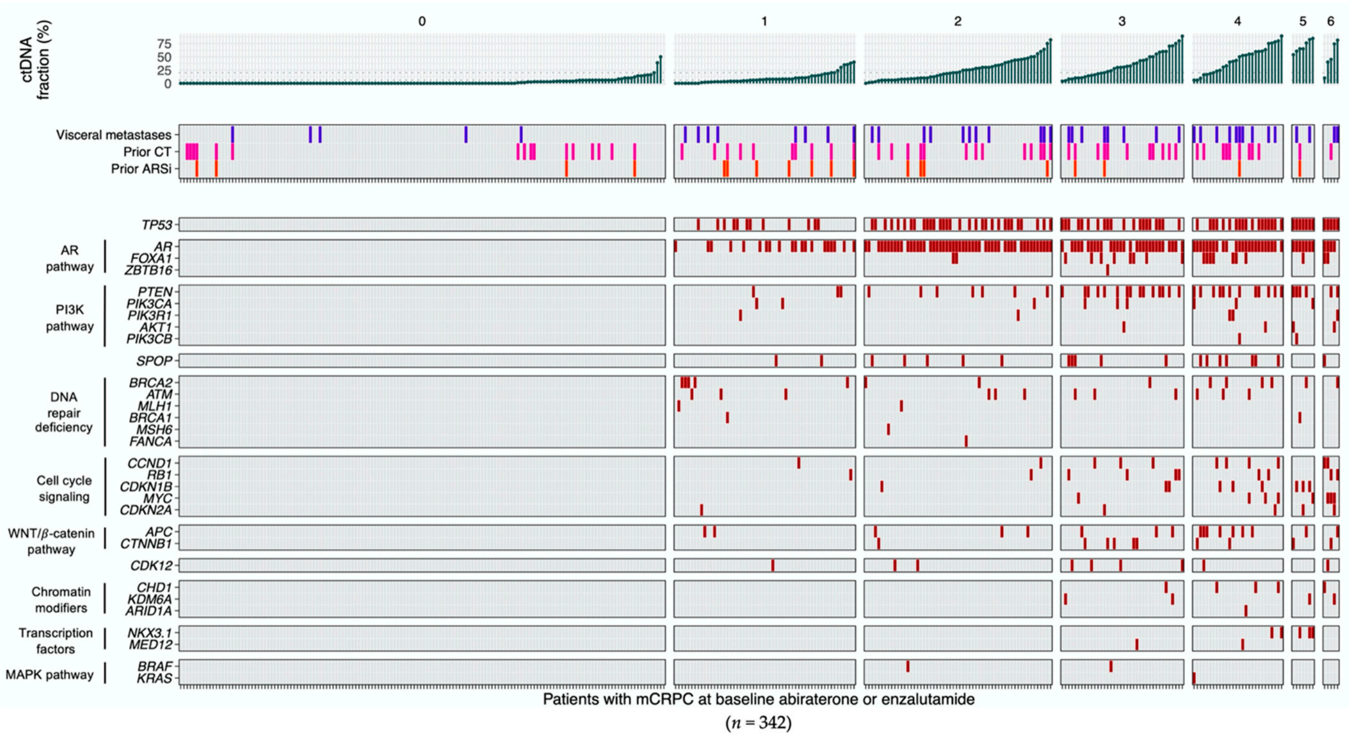

Figure S2. The ctDNA landscape of driver gene perturbations in baseline liquid biopsies from patients with metastatic castration-resistant prostate cancer (n = 342) initiating abiraterone or enzalutamide. Bottom panels: Multi-level landscape of presence (dark red) or absence (grey) of driver gene perturbations, grouped per pathway or gene class (rows). Middle panel: Presence of visceral metastases (blue), and prior chemotherapy (CT, pink) and/or AR signalling inhibitors (ARSi, red) exposure. Top panel: ctDNA fraction (in %). Patients (columns) are grouped according to the number (0 to 6) of perturbed genes that were detected, and ordered according ctDNA fraction. Horizontal dashed lines represent 20% and 6% (i.e. median) ctDNA fraction levels.

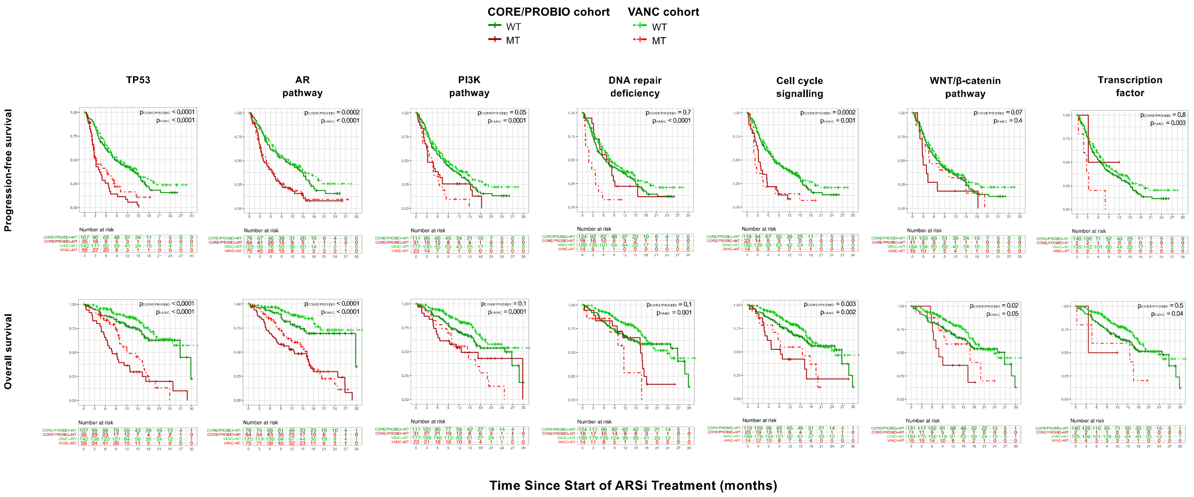

Figure S3. Cohort-stratified Kaplan-Meier analyses (CORE/PROBIO: solid line; VANC: dashed line) of progression-free (upper) and overall (lower) survival, stratified according to a wild-type (WT, i.e. absence of driver) or mutant/perturbed (MT) pathway or gene class status. Only pathways or gene classes with significant associations with PFS and/or OS in univariate analysis on the total patient population (Table S4) were used, i.e. the AR pathway, TP53 class, PI3K pathway, DNA repair deficiency, cell cycle signalling, WNT/β-catenin pathway and transcription factor alterations. *p*-values are calculated via log-rank test.

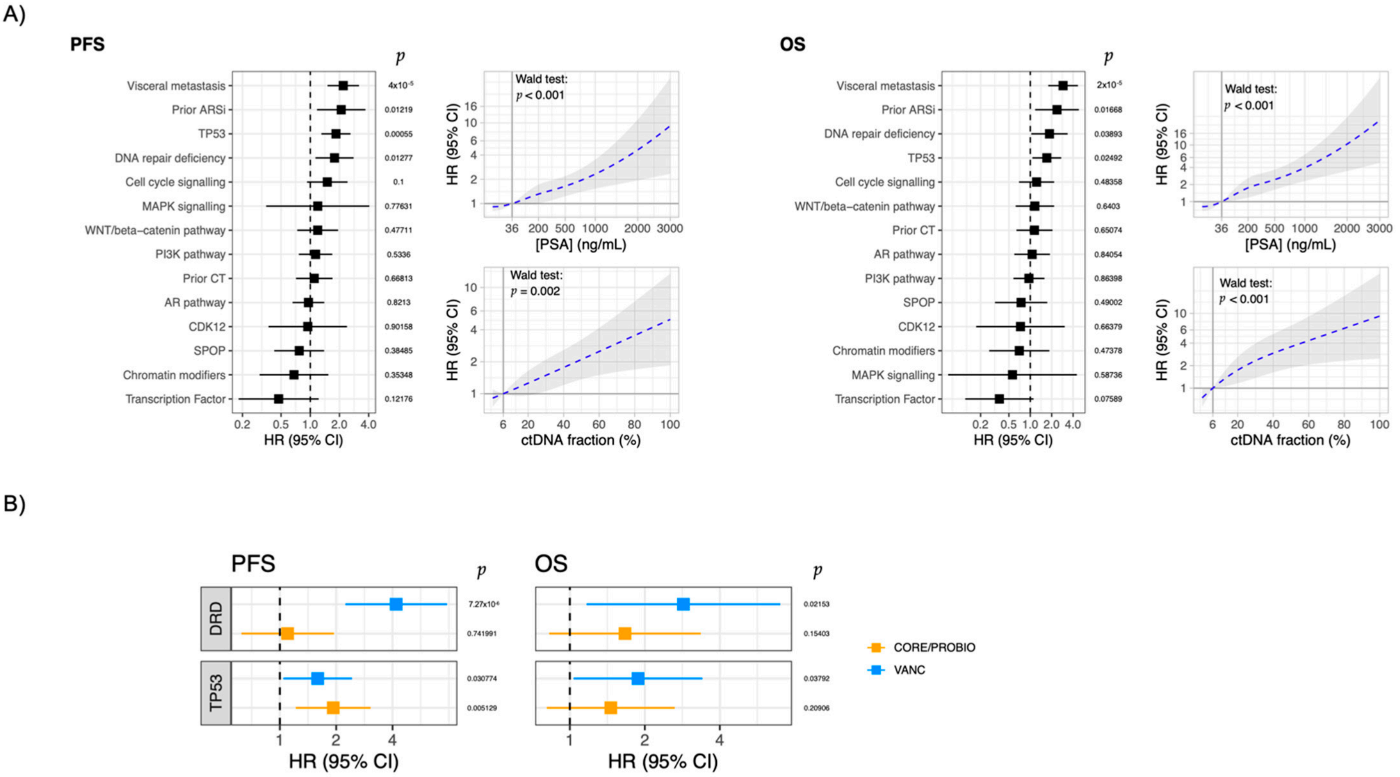

Figure S4. **(A)** Multivariate Cox regression analysis of progression-free (left) and overall (right) survival. Serum PSA and plasma ctDNA were modelled as continuous variables using restricted cubic spline models within the multivariate cox regression models, using the median PSA (36 ng/mL) and ctDNA fraction (6%) as reference (i.e. HR = 1). PFS and OS models use baseline characteristics, together with all pathways or gene classes. **(B)** Multivariate analysis of TP53 and DNA repair categories. Multivariate PFS and OS Cox regression models use baseline characteristics (as described in A), TP53 and DNA repair categories with interaction of the cohort variable. Abbreviations: ARSi, androgen receptor signalling inhibitors; CT, chemotherapy; ctDNA, circulating tumour DNA fraction; PFS, progression-free survival; OS, overall survival; PSA, prostate-specific antigen; HR (95% CI), hazard ratio (95% confidence interval).

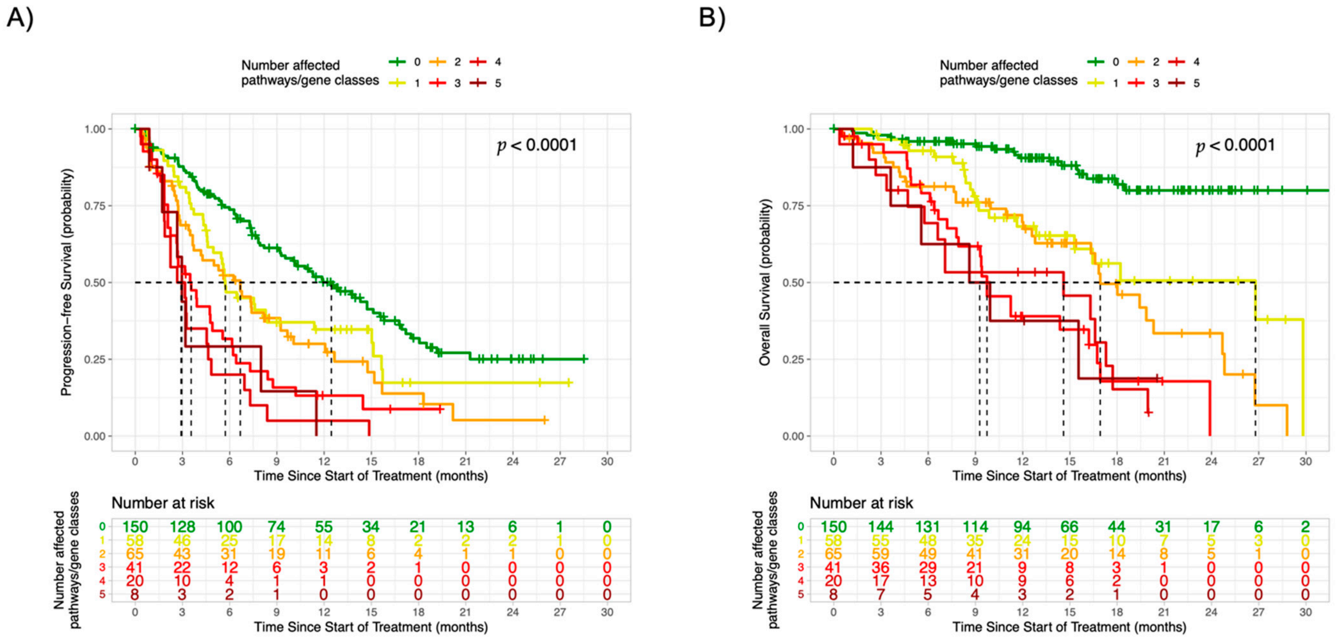

Figure S5. Kaplan-Meier analysis of progression-free survival (A) and overall (B) survival, stratified according to the number of perturbed pathways or gene classes, which demonstrates a decremental effect as the number of affected pathways accumulates. *p*-value is calculated via log-rank test.

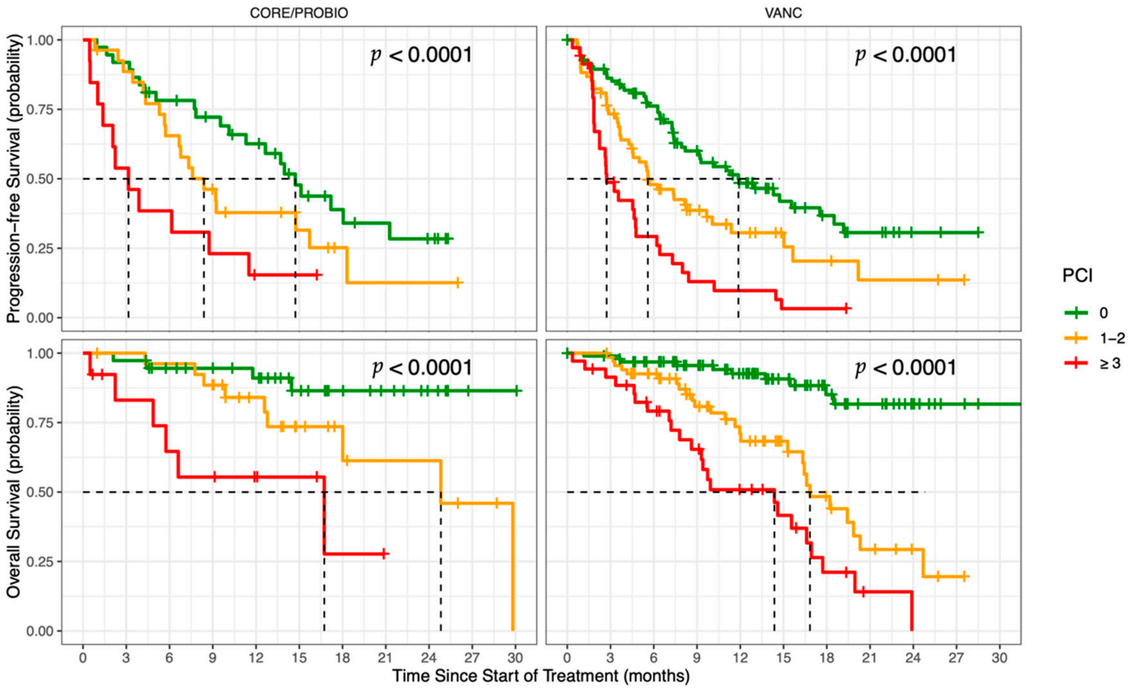

Figure S6. Cohort-stratified Kaplan-Meier analysis of progression-free and overall survival in treatment-naïve patients (i.e. no prior ARSi or CT for mCRPC), stratified according to the Pathway Complexity Index (PCI, i.e. 0, 1–2 and ≥3 perturbed pathways or gene classes). *p*-value is calculated via log-rank test.

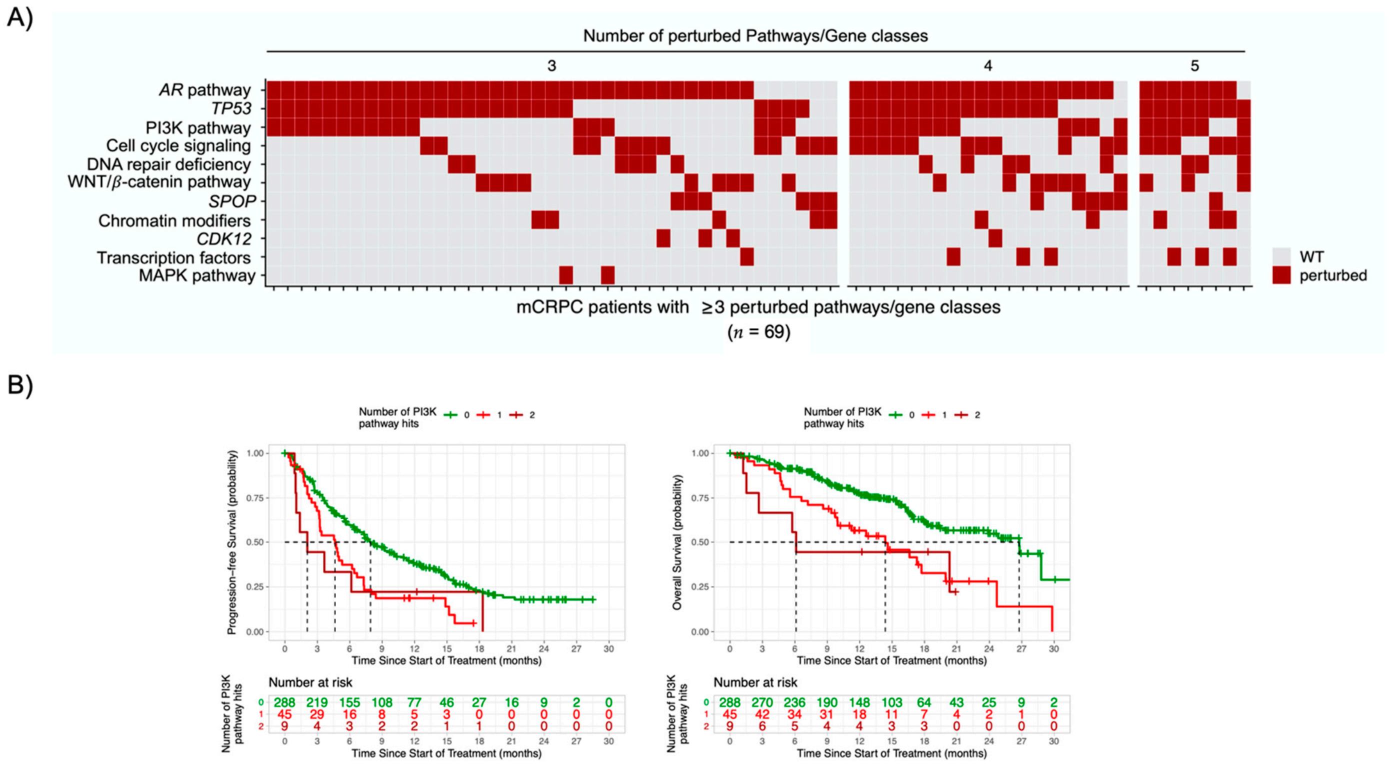

Figure S7. Pathway or gene class characteristics in patients with an elevated pathway complexity index. **(A)** The pathway/gene class landscape in baseline liquid biopsies from ARSi-initiating mCRPC patients with ≥3 affected pathways or gene classes (*n* = 69). Multi-level landscape of presence (dark red) or absence (grey) of pathway/gene class perturbations, grouped per number of pathway or gene class hits. **(B)** PFS and OS prognosis in terms of the number of altered driver genes that overlap within the PI3K pathway.

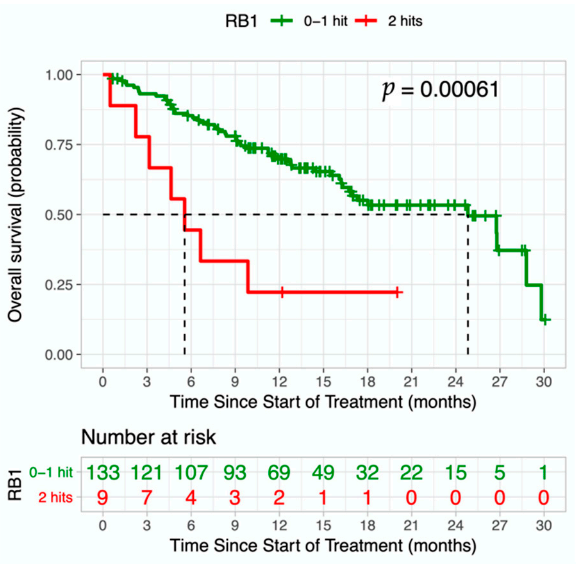

Figure S8. Overall survival between patients with and without biallelic *RB1* inactivation from the date of ARSi initiation.

Table S1. All-comer patient characteristics for the total and separate cohorts. p-values are calculated via Mann-Whitney U and Chi-square tests.

-	Total	CORE/PROBIO	VANC	*p*
N	342	142	200	-
Age (median (IQR))	75 (68.00–81.0)	76.00 (69.00–81.00)	75.00 (68.00–82.00)	0.975
PSA (median (IQR))	36.09 (11.90–120.00	36.09 (13.29–144.70)	36.05 (11.28–107.45)	0.549
ctDNA fraction (median (IQR))	6.00 (0.00–25.00)	9.15 (2.92–30.00)	3.90 (0.00–20.70)	0.001

Prior CT exposure (%)	61 (17.8)	61 (43.0)	0 (0.0)	<0.001
Prior ARSi exposure (%)	19 (5.6)	19 (13.4)	0 (0.0)	<0.001
Visceral metastasis (%)	46 (13.5)	24 (16.9)	22 (11.0)	0.157
Number of perturbed pathways/gene classes (PCI) (%)	-	-	-	0.101
0	150 (43.9)	53 (37.3)	97 (48.5)	-
1–2	123 (36.0)	55 (38.7)	68 (34.0)	-
≥3	69 (20.1)	34 (23.9)	35 (17.5)	-

Table S2. Treatment-naive patient characteristics for the total and separate cohorts. p-values are calculated via Mann-Whitney U and Chi-square tests. CT: chemotherapy, ARSi: AR signaling inhibitor.

-	Total (CT/ARSi-naïve)	CORE/PROBIO	VANC	*p*
N	277	77	200	
Age (median (IQR))	75.00 (68.00–82.00)	78.00 (70.00–82.00)	75.00 (68.00–82.00)	0.250
PSA (median (IQR))	33.45 (10.82 - 104.78)	24.41 (8.00–88.23)	36.05 (11.28–107.45)	0.135
ctDNA fraction (median (IQR))	5.80 (0–20.00)	8.00 (0–20.00)	3.90 (0.00–20.70)	0.108
Visceral metastasis (%)	31 (11.2)	9 (11.7)	22 (11.0)	1.000
Number of perturbed pathways/gene classes (PCI) (%)	-	-	-	0.984
0	134 (48.4)	37 (48.1)	97 (48.5)	-
1–2	95 (34.3)	27 (35.1)	68 (34.0)	-
≥3	48 (17.3)	13 (16.9)	35 (17.5)	-

Table S3. Gene- and Pathway/Gene class-level prevalences of genomic alterations in baseline plasma ctDNA samples from patients with metastatic castration-resistant prostate cancer initiating abiraterone or enzalutamide ($N = 342$).

Gene	# Perturbed Patients (Gene)	Frequency (Gene)	Pathway/Gene Class	# Perturbed Patients (PathWay/Gene Class)	Frequency (Pathway/Gene Class)
AR	135	0.395	*AR* pathway	139	0.406
FOXA1	20	0.058			
ZBTB16	1	0.003			
TP53	93	0.272	*TP53*	93	0.272
PTEN	43	0.126	PI3K pathway	54	0.158
PIK3CA	9	0.026			
PIK3R1	5	0.015			
AKT1	4	0.012			
PIK3CB	2	0.006			
CCND1	11	0.032	Cell cycle signaling	37	0.108
RB1	10	0.029			
CDKN1B	9	0.026			
MYC	8	0.023			
CDKN2A	5	0.015			
CDK4	0	0			
BRCA2	14	0.041	DNA repair deficiency	32	0.094
ATM	12	0.035			
BRCA1	2	0.006			
MLH1	2	0.006			
FANCA	1	0.003			
MSH6	1	0.003			
MSH2	0	0			
APC	17	0.05	WNT/β-catenin pathway	27	0.079
CTNNB1	10	0.029			
SPOP	20	0.058	*SPOP*	20	0.058
CHD1	5	0.015	Chromatin modifiers	10	0.029
KDM6A	4	0.012			
ARID1A	1	0.003			
CDK12	9	0.026	*CDK12*	9	0.026
NKX3.1	5	0.015	Transcription factors	7	0.02

MED12	2	0.006			
NFE2L2	0	0			
BRAF	2	0.006	MAPK pathway	3	0.009
KRAS	1	0.003			
IDH1	0	0	*IDH1*	0	0
IDH2	0	0	Other	0	0
FBXW7	0	0			
GNAS	0	0			
FANCG	0	0			

Table S4. Comparative analysis of the prevalence of the top 5 most commonly perturbed genes between the CORE/PROBIO (*N* = 142) and VANC (*N* = 200) cohorts. *p*-values are calculated via Chi-square test.

-	Total	CORE/PROBIO	VANC	*p*
N	342	142	200	-
AR = perturbed (%)	135 (39.5)	63 (44.4)	72 (36.0)	0.148
TP53 = perturbed (%)	93 (27.2)	35 (24.6)	58 (29.0)	0.442
PTEN = perturbed (%)	43 (12.6)	28 (19.7)	15 (7.5)	0.001
FOXA1 = perturbed (%)	20 (5.8)	6 (4.2)	14 (7.0)	0.399
SPOP = perturbed (%)	20 (5.8)	8 (5.6)	12 (6.0)	1.000

Table S5. Kaplan-Meier analyses, and uni- and multivariate Cox regression analyses (hazard ratio (confidence interval)) of progression-free survival, stratified according to a wild-type (WT, i.e. absence of driver alteration) or perturbed pathway/gene class status. *p*-values are calculated via log-rank test and Wald tests. # denotes that each pathway/gene class is entered individually in a multivariate Cox regression model, including the following covariates: baseline PSA and ctDNA levels, prior chemotherapy, prior ARSi and presence of visceral metastases.

-				Univariate		Multivariate #		-
**Pathway/Gene Class**	***N* (WT vs Perturbed)**	**KM Estimates (In Months)**	***p***	**Cox PH HR (95%CI)**	***p***	**Cox PH HR (95%CI)**	***p***	-
AR pathway	224 vs 139	10.2 vs 4.6	<0.0001	2.1 (1.6–2.7)	< 0.0001	1.1 (0.8–1.5)	0.7	-
TP53	249 vs 93	9.2 vs 3.2	<0.0001	2.6 (2.0–3.5)	< 0.0001	1.7 (1.2–2.3)	0.002	**
SPOP	322 vs 20	7.3 vs 6.7	0.63	1.13 (0.7–1.9)	0.6	0.7 (0.4–1.2)	0.2	-
PI3K pathway	288 vs 54	7.9 vs 4.5	<0.0001	1.9 (1.4–2.7)	< 0.0001	1.2 (0.8–1.7)	0.3	-
Cell cycle signaling	305 vs 37	7.7 vs 3.2	<0.0001	2.4 (1.7–3.5)	< 0.0001	1.3 (0.8–2.0)	0.3	-
WNT/β-catenin pathway	315 vs 27	7.4 vs 4.3	0.07	1.5 (0.9–2.3)	0.07	1.0 (0.6–1.6)	0.9	-
DNA repair deficiency	310 vs 32	7.4 vs 4.6	0.005	1.8 (1.2–2.6)	0.006	1.6 (1.03–2.4)	0.04	*
CDK12	333 vs 9	7.3 vs 8.2	0.6	1.3 (0.6–2.9)	0.6	0.7 (0.3–1.5)	0.3	-
Chromatin modifiers	332 vs 10	7.3 vs 3.6	0.3	1.4 (0.7–2.9)	0.3	0.6 (0.3–1.4)	0.3	-
Transcription factors	335 vs 7	7.3 vs 3.2	0.05	2.2 (0.9–4.9)	0.06	0.7 (0.3–1.8)	0.5	-
MAPK pathway	339 vs 3	7.3 vs 6.2	0.37	1.7 (0.5–5.2)	0.4	1.1 (0.3–3.5)	0.9	-

Table S6. Kaplan-Meier analyses, and uni- and multivariate Cox regression analyses (hazard ratio (confidence interval)) of overall survival, stratified according to a wild-type (WT, i.e. absence of driver alteration) or perturbed pathway/gene class status. *p*-values are calculated via log-rank test and Wald tests. # denotes that each pathway/gene class is entered individually in a multivariate Cox regression model, including the following covariates: baseline PSA and ctDNA levels, prior chemotherapy, prior ARSi and presence of visceral metastases.

-				Univariate		Multivariate #		-
**Pathway/Gene class**	***N* (WT vs Perturbed)**	**KM Estimates (In Months)**	***p***	**Cox PH HR (95%CI)**	***p***	**Cox PH HR (95%CI)**	***p***	-
AR pathway	224 vs 139	29.8 vs 15.5	<0.0001	4.2 (2.9–6.3)	< 0.0001	1.1 (0.7–1.9)	0.6	-
TP53	249 vs 93	29.8 vs 10.9	<0.0001	4.0 (2.8–5.8)	< 0.0001	1.6 (1.0–2.4)	0.04	*
SPOP	322 vs 20	24.7 vs 20.0	0.8	1.1 (0.5–2.4)	0.8	0.7 (0.3–1.4)	0.3	-

PI3K pathway	288 vs 54	26.8 vs 14.4	<0.0001	2.3 (1.6–3.5)	< 0.0001	1.0 (0.7–1.6)	0.9	-
Cell cycle signaling	305 vs 37	24.8 vs 9.9	<0.0001	2.8 (1.8–4.4)	< 0.0001	1.1 (0.6–1.8)	0.9	-
WNT/β-catenin pathway	315 vs 27	24.8 vs 9.9	0.002	2.3 (1.3–3.9)	0.002	1.2 (0.7–2.1)	0.6	-
DNA repair deficiency	310 vs 32	24.8 vs 16.3	0.001	2.3 (1.4–3.8)	0.001	1.8 (1.0–3.1)	0.04	*
CDK12	333 vs 9	23.9 vs 6.8	0.4	1.7 (0.5–5.4)	0.4	0.7 (0.2–2.3)	0.5	-
Chromatin modifiers	332 vs 10	24.7 vs 14.6	0.09	2.0 (0.9–4.6)	0.09	0.6 (0.2–1.5)	0.3	-
Transcription factors	335 vs 7	24.7 vs 15.5	0.04	2.5 (1.0–6.3)	0.04	0.5 (0.2–1.4)	0.2	-
MAPK pathway	339 vs 3	23.9 vs 26.8	0.7	0.6 (0.1–4.6)	0.7	0.5 (0.1–3.6)	0.5	-

Publisher's Note: MDPI stays neutral with regard to jurisdictional claims in published maps and institutional affiliations.

© 2021 by the authors. Licensee MDPI, Basel, Switzerland. This article is an open access article distributed under the terms and conditions of the Creative Commons Attribution (CC BY) license (http://creativecommons.org/licenses/by/4.0/).
